# Phase-Dependent Changes in Heart Rate Variability During the Menstrual Cycle in Healthy Young Women at a Tertiary Care Center

**DOI:** 10.7759/cureus.113483

**Published:** 2026-07-27

**Authors:** Krity Singh, Vishavdeep Kaur, Ankalayya Bobbara

**Affiliations:** 1 Department of Physiology, Maharishi Markandeshwar Institute of Medical Sciences and Research, Ambala, IND

**Keywords:** autonomic nervous system, heart rate variability, menstrual cycle, ovulatory phase, parasympathetic activity, sympathetic activity

## Abstract

Background

The menstrual cycle is characterized by cyclical hormonal fluctuations of estrogen and progesterone that influence multiple physiological systems, including the cardiovascular and autonomic nervous systems. Heart rate variability (HRV) is a widely accepted, non-invasive method for assessing autonomic nervous system activity. This study aimed to evaluate HRV across different phases of the menstrual cycle in young, healthy female students.

Methodology

This observational study was conducted in the Department of Physiology at Maharishi Markandeshwar Institute of Medical Sciences and Research (MMIMSR), Mullana, Ambala, over 18 months. Fifty healthy female students aged 18-30 years with regular menstrual cycles were included. HRV was recorded using electrocardiography during the menstrual, proliferative, ovulatory, and secretory phases. Frequency-domain parameters were analyzed using SPSS software (IBM SPSS Statistics for Windows, IBM Corp., Version 23.0, Armonk, NY).

Results

Fifty healthy women (mean age: 20.3 ± 2.6 years) were included. High-frequency (HF) power varied significantly across menstrual phases (F = 15.28, p < 0.001), increasing from 807 ± 949 ms² during the menstrual phase to 2118 ± 2013 ms² in the proliferative phase and 1753 ± 1498 ms² during ovulation before decreasing to 912 ± 1194 ms² in the secretory phase. Low-frequency (LF) power also showed significant phase-dependent variation (F = 8.48, p < 0.001), whereas the LF/HF ratio did not differ significantly between phases (F = 0.74, p = 0.529). Post hoc analysis demonstrated significantly higher HF power during the proliferative and ovulatory phases compared with the menstrual and secretory phases (all p ≤ 0.002), indicating greater parasympathetic activity during estrogen-dominant phases.

Conclusion

HRV demonstrates phase-dependent variation across the menstrual cycle, with increased parasympathetic activity during the proliferative and ovulatory phases. Consideration of the menstrual phase is important when interpreting HRV findings.

## Introduction

The menstrual cycle is a complex physiological process regulated by the hypothalamic-pituitary-ovarian axis. Hormones such as follicle-stimulating hormone, luteinizing hormone, estrogen, and progesterone coordinate cyclic ovarian activity and endometrial changes throughout the reproductive years in women. These hormonal fluctuations influence several systemic physiological processes, including cardiovascular function and autonomic nervous system regulation [1-3].

Heart rate variability (HRV) represents beat-to-beat variations in heart rate and reflects the dynamic interaction between the sympathetic and parasympathetic components of the autonomic nervous system. It is widely used as a non-invasive tool for assessing autonomic function. Frequency-domain analysis of HRV provides insight into cardiac autonomic modulation through low-frequency (LF) and high-frequency (HF) components [4].

Estrogen is associated with enhanced parasympathetic activity and vasodilatory effects, whereas progesterone is linked with increased sympathetic activity. Therefore, autonomic regulation of cardiovascular function may vary across different phases of the menstrual cycle [2,5].

Previous studies have reported phase-dependent variations in heart rate variability (HRV); however, the findings remain inconsistent [6-10]. Therefore, the present study aimed to compare frequency-domain HRV parameters (HF power, LF power, and LF/HF ratio) across the menstrual, proliferative, ovulatory, and secretory phases in healthy young women using a repeated-measures design. We hypothesized that parasympathetic activity, reflected by higher HF power, would be greater during the estrogen-dominant proliferative and ovulatory phases than during the menstrual and secretory phases. The primary outcome measure was HF power, while LF power and the LF/HF ratio were evaluated as secondary outcome measures.

## Materials and methods

Study design and setting

This cross-sectional observational study was conducted in the Department of Physiology at Maharishi Markandeshwar Institute of Medical Sciences and Research (MMIMSR), Mullana, Ambala, Haryana, from 18 February 2025 to 18 February 2026. Written informed consent was obtained from all participants before enrollment. Confidentiality and anonymity were strictly maintained throughout the study in accordance with ethical research standards.

Study population

A total of 50 healthy female students aged 18-30 years were included. Participants were recruited from the institutional student population using convenience sampling. Only individuals with regular menstrual cycles were included to ensure reliable phase-wise comparison of autonomic parameters.

Inclusion and exclusion criteria

Healthy females within the specified age group with regular menstrual cycles ranging from 26 to 32 days were included in the study. Participants with irregular menstrual cycles or with a history of cardiovascular, endocrine, neurological, or metabolic disorders were excluded. Individuals on medications affecting autonomic function were also excluded. In addition, users of hormonal contraceptives and those with a history of smoking, alcohol consumption, excessive caffeine intake, recent acute illness, or significant psychological stress were excluded to minimize confounding effects on HRV.

Sample size calculation

The sample size for the study was calculated using the formula for comparing means across repeated measurements:

\[ n=\frac{\left(Z_{1-\alpha/2}+Z_{1-\beta}\right)^2\times\sigma^2\times r} {\displaystyle\sum_{i=1}^{r}(\mu_i-\mu)^2} \]

where n is the required sample size, Z₁−α/₂ is the standard normal deviate corresponding to the chosen level of significance, Z₁−β is the standard normal deviate corresponding to the desired statistical power, σ² is the variance of the outcome variable, r is the number of repeated measurements, μᵢ is the mean value of the outcome at the repeated measurement, and μ is the overall mean of the outcome variable.

The values required for the sample size calculation were obtained from the study by Hamidovic et al. [11], which reported the variance and phase-specific mean values of HF power among healthy women across the menstrual cycle. The parameters used were as follows: α = 0.05, power = 80% (*β=0.20*; $Z_{1-\beta}=0.84$), variance of HF power σ² = 1,200,000 ms², number of repeated measurements *r=4*, phase-specific mean HF power values of 850, 2200, 1800, and 950 ms² during the menstrual, proliferative, ovulatory, and secretory phases, respectively, and an overall mean HF power of 1450 ms².

Substituting these values into the formula:

\[ n= \frac{(1.96+0.84)^2\times1,200,000\times4} {(850-1450)^2+(2200-1450)^2+(1800-1450)^2+(950-1450)^2} \approx45 \]

Accordingly, the minimum required sample size was 45 participants. To account for potential dropouts and ensure adequate statistical power, a total of 50 participants were enrolled in the study.

Assessment of menstrual cycle phases

Menstrual cycle phases were determined using self-reported menstrual history and the calendar method. The menstrual phase was defined as days 1-5, the proliferative (follicular) phase as days 6-13, the ovulatory phase as day 14 ± 1, and the secretory (luteal) phase as days 15-28. Participants maintained menstrual cycle records before data collection to improve the accuracy of phase identification.

Anthropometric and baseline measurements

Baseline anthropometric parameters, including height and weight, were measured using standard instruments. Body mass index (BMI) was calculated as weight in kilograms divided by height in meters squared. Resting heart rate and blood pressure were recorded before HRV assessment after participants had been seated comfortably for at least five minutes.

HRV recording

HRV was recorded using a PhysioTel digital data-acquisition system (Data Sciences International, New Brighton, MN). Recordings were performed in a quiet, temperature-controlled laboratory environment maintained at 22°C-24°C to minimize external influences. Participants were instructed to avoid caffeine, heavy meals, and strenuous physical activity for at least 12 hours before recording. Each participant rested in the supine position for ten minutes before data acquisition to ensure cardiovascular stabilization. A continuous 10-minute electrocardiographic recording was then obtained during each phase of the menstrual cycle. Each participant underwent HRV assessment during all four menstrual cycle phases, and repeated measurements were obtained from the same participants throughout the study period.

HRV analysis

Electrocardiographic data were analyzed using frequency-domain methods to assess autonomic function. LF and HF power were calculated in ms². LF power reflects combined sympathetic and parasympathetic activity, whereas HF power primarily represents parasympathetic (vagal) activity. Data were visually inspected, and artifacts and ectopic beats were removed before analysis to ensure accuracy and reliability.

Ethical approval

The study protocol was reviewed and approved by the Institutional Ethics Committee of Maharishi Markandeshwar Institute of Medical Sciences and Research, Mullana, Ambala (Approval No.: IEC-3284). Written informed consent was obtained from all participants before enrollment. The study was conducted in accordance with the ethical principles of the Declaration of Helsinki.

Statistical analysis

All data were entered and analyzed using Statistical Package for the Social Sciences (SPSS) (IBM SPSS Statistics for Windows, IBM Corp., Version 23.0, Armonk, NY). Continuous variables were expressed as mean and standard deviation. The normality of data distribution was assessed using the Shapiro-Wilk test. To compare HRV parameters (VLF power, LF power, HF power, LF/HF ratio, LF norm, and HF norm) across the different phases of the menstrual cycle, a repeated measures analysis of variance (ANOVA) was employed. Where appropriate, post hoc analysis with Bonferroni correction was applied to identify specific inter-phase differences. Pearson's correlation analysis was performed to assess the relationship between HRV parameters and cardiovascular variables (resting heart rate, systolic blood pressure, diastolic blood pressure), as well as between BMI and HF power across different menstrual phases. A p-value of less than 0.05 was considered statistically significant.

## Results

The participants were young, healthy women with a mean age of 20.3 ± 2.6 years. The mean BMI was 22.3 ± 1.5 kg/m², indicating normal anthropometric characteristics among most subjects (Table 1).

**Table 1:** Baseline characteristics of participants (n = 50)

Parameter	Mean ± SD
Age (years)	20.3 ± 2.6
Height (cm)	162.5 ± 2.8
Weight (kg)	59.2 ± 4.9
BMI (kg/m²)	22.3 ± 1.5

The mean resting heart rate was 109.7 ± 28.4 beats per minute (bpm). In contrast, the mean systolic and diastolic blood pressures were 114.3 ± 7.2 mmHg and 73.6 ± 5.6 mmHg, respectively, indicating a normal cardiovascular profile in the study population (Table 2).

**Table 2:** Baseline cardiovascular parameters (n = 50)

Parameter	Mean ± SD
Resting heart rate (bpm)	109.7 ± 28.4
Systolic BP (mmHg)	114.3 ± 7.2
Diastolic BP (mmHg)	73.6 ± 5.6

Table 3 demonstrates statistically significant variation in both LF power and HF power across different phases of the menstrual cycle. LF power showed significant variation (F = 8.48, p < 0.001), while HF power demonstrated highly significant variation (F = 15.28, p < 0.001). HF power increased from 807 ± 949 ms² in the menstrual phase to 2118 ± 2013 ms² in the proliferative phase and remained relatively high during ovulation (1753 ± 1498 ms²) before decreasing during the secretory phase (912 ± 1194 ms²). The LF/HF ratio showed a decreasing trend across phases but did not reach statistical significance (F = 0.74, p = 0.529).

**Table 3:** HRV spectral power and LF/HF ratio across menstrual phases with repeated measures ANOVA results *Indicates statistically significant difference (p < 0.05). Analysis performed using repeated measures ANOVA. LF, low frequency; HF, high frequency; HRV, heart rate variability

Parameter	Menstrual phase (mean ± SD)	Proliferative phase (mean ± SD)	Ovulatory phase (mean ± SD)	Secretory phase (mean ± SD)	F-value	p-value
LF power (ms²)	1011 ± 1564	1500 ± 1117	1213 ± 853	799 ± 1015	8.48	<0.001*
HF power (ms²)	807 ± 949	2118 ± 2013	1753 ± 1498	912 ± 1194	15.28	<0.001*
LF/HF ratio	1.88 ± 1.56	1.29 ± 0.95	1.12 ± 0.89	1.20 ± 0.73	0.74	0.529

Post hoc analysis revealed that HF power during the proliferative phase was significantly higher than the menstrual phase (mean difference = -1311.17, p < 0.001) and the secretory phase (mean difference = 1205.94, p = 0.002). Similarly, HF power during the ovulatory phase was significantly higher compared to the menstrual phase (mean difference = -945.70, p = 0.002) and the secretory phase (mean difference = 840.47, p = 0.002). No statistically significant difference was observed between the menstrual and secretory phases (mean difference = -105.24, p = 1.000). These findings suggest enhanced parasympathetic activity during estrogen-dominant phases of the menstrual cycle (Table 4).

**Table 4:** Post hoc Bonferroni analysis for HF power across menstrual phases *Indicates statistically significant difference (p < 0.05) after Bonferroni correction. Analysis performed using repeated measures ANOVA with Bonferroni correction for multiple comparisons. HF, high frequency

Comparison (Phase I vs. Phase II)	Mean difference (I-II)	Standard error	p-value
Menstrual vs. proliferative	-1311.17	245.3	<0.001*
Menstrual vs. ovulatory	-945.70	231.8	0.002*
Menstrual vs. secretory	-105.24	186.4	1.000
Proliferative vs. ovulatory	365.47	218.6	0.356
Proliferative vs. secretory	1205.94	262.1	0.002*
Ovulatory vs. secretory	840.47	244.7	0.002*

Table 5 presents the post hoc analysis for LF power. LF power was significantly higher in the proliferative phase compared to the menstrual phase (mean difference = -489.00, p = 0.028) and secretory phase (mean difference = 701.00, p < 0.001). LF power was also significantly higher in the ovulatory phase compared to the secretory phase (mean difference = 414.00, p = 0.011). Additionally, LF power was significantly higher in the proliferative phase compared to the ovulatory phase (mean difference = 287.00, p = 0.047). No statistically significant differences were observed between the menstrual and ovulatory phases (p = 0.120) or between the menstrual and secretory phases (p = 0.156). These findings indicate that LF power shows significant phase-dependent variation, with higher values during the proliferative phase and lower values during the secretory phase.

**Table 5:** Post hoc Bonferroni analysis for LF power across menstrual phases *Indicates statistically significant difference (p < 0.05) after Bonferroni correction. Analysis performed using repeated measures ANOVA with Bonferroni correction for multiple comparisons. LF, low frequency

Comparison (Phase I vs. Phase II)	Mean difference (I-II)	Standard error	p-value
Menstrual vs. proliferative	-489.00	212.4	0.028*
Menstrual vs. ovulatory	-202.00	127.6	0.120
Menstrual vs. secretory	212.00	145.7	0.156
Proliferative vs. ovulatory	287.00	139.5	0.047*
Proliferative vs. secretory	701.00	184.9	<0.001*
Ovulatory vs. secretory	414.00	152.3	0.011*

Table 6 demonstrates that the LF/HF ratio was highest during the menstrual phase (3.16 ± 10.84) and progressively decreased during the proliferative (1.29 ± 0.90), ovulatory (1.26 ± 1.92), and secretory phases (1.15 ± 0.34). These findings indicate a relative improvement in sympathovagal balance during proliferative and ovulatory phases.

**Table 6:** HRV spectral power (absolute and normalized) across menstrual phases LF norm = (LF power/(LF power + HF power)) × 100; HF norm = (HF power/(LF power + HF power)) × 100. The values are expressed in normalized units (n.u.), which are dimensionless ratios. LF, low frequency; HF, high frequency; HRV, heart rate variability

Phase	LF/HF ratio (mean ± SD)	LF norm (n.u.) (mean ± SD)	HF norm (n.u.) (mean ± SD)
Menstrual	1.88 ± 1.56	53.68 ± 14.2	46.23 ± 13.1
Proliferative	1.29 ± 0.95	49.43 ± 14.2	50.52 ± 13.8
Ovulatory	1.12 ± 0.89	45.53 ± 13.4	54.47 ± 13.4
Secretory	1.20 ± 0.73	51.23 ± 12.5	48.77 ± 12.5

HF power showed a significant negative correlation with resting heart rate (r = -0.32, p = 0.02), while LF/HF ratio demonstrated a significant positive correlation with resting heart rate (r = 0.41, p = 0.003). No significant correlations were observed between HRV parameters and blood pressure measurements (Table 7).

**Table 7:** Pearson's correlation analysis between HRV parameters and cardiovascular variables Pearson's correlation coefficient (r) was used to assess the linear relationship between variables. LF, low frequency; HF, high frequency; HRV, heart rate variability

Parameter	Resting heart rate (r, p-value)	Systolic BP (r, p-value)	Diastolic BP (r, p-value)
HF power (ms²)	-0.32, 0.02	0.12, 0.39	0.06, 0.68
LF/HF ratio	0.41, 0.003	-0.08, 0.58	-0.11, 0.44

Table 8 shows weak, statistically nonsignificant correlations between BMI and HF power across all menstrual phases. Pearson correlation coefficients were 0.170 during the menstrual phase (p = 0.238), -0.147 during the proliferative phase (p = 0.308), -0.182 during the ovulatory phase (p = 0.207), and -0.184 during the secretory phase (p = 0.201). These findings suggest that body mass index had no significant influence on HF power in the present study population, and the observed HRV variations were more likely associated with cyclical hormonal fluctuations.

**Table 8:** Correlation of BMI with HF power during different menstrual phases Pearson's correlation coefficient (r) was used to assess the linear relationship between BMI and HF power. HF, high frequency

Menstrual phase	Correlation coefficient (r)	p-value
Menstrual	0.170	0.238
Proliferative	-0.147	0.308
Ovulatory	-0.182	0.207
Secretory	-0.184	0.201

## Discussion

The present study demonstrated variation in HRV parameters across different phases of the menstrual cycle, indicating that fluctuations in ovarian hormones influence autonomic nervous system activity. The study population consisted of young healthy women with a mean age of 20.3 ± 2.6 years and a mean BMI of 22.3 ± 1.5 kg/m². The relatively homogeneous age group and normal BMI helped minimize potential confounding factors such as age-related autonomic decline and obesity-related alterations in HRV. Similar observations regarding the influence of age and BMI on HRV have been reported by Vallejo et al. [6].

In the present study, HF power was higher during the proliferative and ovulatory phases, indicating enhanced parasympathetic activity during these phases. Estrogen levels progressively rise during the proliferative phase and peak around ovulation, and are known to enhance vagal activity and improve autonomic balance. These findings are consistent with those of Leicht et al. [7], who reported a positive association between endogenous estrogen levels and HRV parameters during the menstrual cycle. The increased parasympathetic activity observed during these phases suggests a period of relatively favorable autonomic balance in healthy young women.

During the menstrual phase, HF power was comparatively lower than in the proliferative and ovulatory phases, suggesting reduced parasympathetic activity. This may be attributed to the withdrawal of ovarian hormones during menstruation. Similar findings have been reported by Sato et al. [8], who demonstrated phase-dependent variation in autonomic modulation during the menstrual cycle. Dimitriev et al. [9] also reported increased sympathetic influence during phases associated with lower ovarian hormone levels.

The proliferative phase showed a marked increase in HF power compared to the menstrual phase, indicating progressive enhancement of parasympathetic activity. This pattern corresponds with rising estrogen levels during this phase. Comparable findings have been reported by Leicht et al. [7] and Brar et al. [10], who observed improved autonomic balance during the follicular or proliferative phase.

In the secretory phase, HF power declined compared to the ovulatory phase, suggesting a relative reduction in parasympathetic activity. This may be due to progesterone's association with increased sympathetic activity and reduced vagal tone. Yildirir et al. [12] reported similar findings, demonstrating increased sympathetic predominance during the luteal phase compared to the follicular phase. Schmalenberger et al. [13] also observed that progesterone is negatively associated with vagally mediated HRV across the menstrual cycle.

Tenan et al. [14] reported changes in resting HRV across the menstrual cycle, supporting the role of ovarian hormones in autonomic regulation. However, some studies, including Washio et al. [15] and Tousignant-Laflamme et al. [16], have reported inconsistent or minimal variations in HRV across menstrual phases. These discrepancies may be attributed to methodological differences, variations in sample characteristics, or differences in study design. In the present study, standardized short-term HRV recordings under controlled laboratory conditions may have improved the detection of phase-wise autonomic variations.

Overall, the findings of the present study support the hypothesis that estrogen enhances parasympathetic activity, whereas progesterone contributes to relative sympathetic predominance. These hormonal influences result in significant variation in HRV across the menstrual cycle. The results highlight the importance of considering menstrual cycle phase when interpreting HRV in both clinical and research settings.

Limitations

The present study has certain limitations. First, the sample size was relatively small and included participants from a single institution, which may limit the generalizability of the findings. Second, menstrual cycle phases were identified using the calendar method rather than hormonal assays, which may have introduced minor inaccuracies in phase determination. Third, only frequency-domain HRV parameters were evaluated, while time-domain and non-linear HRV indices were not assessed. Future studies involving larger populations, hormonal profiling, and comprehensive autonomic assessment may provide more robust evidence regarding autonomic variations across the menstrual cycle.

Recommendation

Based on the findings of the present study, it is recommended that the menstrual cycle phase be considered when evaluating HRV in women. Since significant variations in autonomic modulation were observed across phases, particularly increased parasympathetic activity during the proliferative and ovulatory phases, HRV assessment in female subjects should ideally be standardized by menstrual cycle phase to ensure accurate interpretation.

Future studies with larger sample sizes and the inclusion of hormonal assays are recommended to further elucidate the relationship between ovarian hormones and autonomic function. Additionally, longitudinal studies involving diverse populations may help establish normative HRV reference values for different phases of the menstrual cycle.

## Conclusions

The present study demonstrates that HRV exhibits phase-dependent variation across the menstrual cycle in healthy young women. Increased HF power during the proliferative and ovulatory phases indicates enhanced parasympathetic activity, whereas relatively lower HF power during the menstrual and secretory phases suggests altered autonomic balance. These findings are consistent with an association between menstrual cycle phase and autonomic modulation, with increased parasympathetic activity observed during the proliferative and ovulatory phases. Although the observed pattern is compatible with the influence of ovarian hormonal fluctuations, hormone concentrations were not directly measured; therefore, causal inferences cannot be established from this observational study. Consideration of menstrual cycle phase remains important when interpreting HRV findings in both clinical and research settings. Accordingly, consideration of the menstrual cycle phase is essential when interpreting HRV in women in both clinical and research settings.
